# Troxerutin Potentiated Temozolomide Induced Antitumor Effect in 2D and 3D Glioblastoma Models

**DOI:** 10.1111/jcmm.70862

**Published:** 2025-10-03

**Authors:** Deborah Mannino, Elsa Calcaterra, Sarah Adriana Scuderi, Maria Caffo, Fabiola De Luca, Antonio Catalfamo, Giovanna Casili, Irene Paterniti

**Affiliations:** ^1^ Research Operative Unit of Neuropharmacology and Translational Neurosciences Oasi Research Institute Troina Italy; ^2^ Department of Chemical, Biological, Pharmaceutical and Environmental Science University of Messina Messina Italy; ^3^ Unit of Neurosurgery, Department of Biomedical and Dental Sciences and Morphofunctional Imaging University of Messina Messina Italy

**Keywords:** glioblastoma, natural flavonoid, oxidative stress, temozolomide, troxerutin

## Abstract

Glioblastoma (GBM) is a highly aggressive brain tumour characterised by rapid proliferation and high invasiveness. Temozolomide (TMZ) is the first‐line chemotherapy, but its efficacy is often compromised by intrinsic or acquired resistance and significant side effects. Recent studies have demonstrated that combining TMZ with natural compounds can enhance therapeutic efficacy through synergistic mechanisms and reduce systemic toxicity. In this study, the adjuvant potential of troxerutin (TROX), a natural flavonoid present found in tea, coffee, cereals, fruits and vegetables, and known for its antioxidant and anti‐inflammatory properties, was evaluated in an in vitro model of human U87 cells. The TROX–TMZ combination showed a significant synergistic effect, effectively reducing cell viability, clonogenic capacity, migration and epithelial‐mesenchymal transition (EMT), as well as promoting apoptosis. The combined treatment also modulated oxidative stress and inflammation by activating the KEAP1/NRF2 axis, resulting in increased levels of HO‐1, GSH and SOD1, and decreased ROMO1, ROS, nitrites and pro‐inflammatory cytokines (IL‐6, TNF‐α and IL‐1β). These effects were also confirmed in three‐dimensional models (spheroids), suggesting that the combination exerts significant antitumor activity. These results indicate that TROX could represent an effective adjuvant in the treatment of GBM, providing a novel therapeutic strategy to potentiate the antitumor effects of TMZ.

## 1 Introduction

Glioblastoma (GBM) accounts for approximately 50% of primary malignant tumours of the central nervous system [1]. Among the most widely used chemotherapeutic agents in the treatment of GBM, temozolomide (TMZ) is currently the standard first‐line therapy. TMZ is an oral alkylating agent that induces by inducing DNA lesions, particularly at guanine residues, thereby causing genomic instability and leading to apoptosis of tumour cells. However, treatment with TMZ is often associated with a series of side effects, including nausea, vomiting, asthenia, myelosuppression and hepatotoxicity [2]. In recent years, several epidemiological studies have highlighted the potential role of natural compounds in modulating the development, progression and dissemination across different cancer types [3, 4, 5]. Preclinical evidence suggests that combining TMZ with natural compounds may enhance its antiproliferative effects, indicating a potential synergistic interaction that could pave the way for the development of more effective and better‐tolerated combination therapies for the treatment of GBM [6, 7]. Moreover, excessive reactive oxygen species (ROS) production in tumours, including GBM, leads to an overload of DNA repair mechanisms and may compromise membrane integrity and protein stability, thus contributing to tumour progression [8]. In this context, the integration of complementary therapeutic approaches, such as the use of natural antioxidant compounds or other innovative strategies, may not only potentiate the antitumour efficacy of TMZ but also mitigate treatment‐related side effects [9, 10, 11, 12]. Recent scientific evidence has suggested that Troxerutin (TROX) exerts antitumour, anti‐inflammatory and antioxidant effects [13, 14, 15]. TROX is a natural flavonoid belonging to the rutin class, mainly extracted from *Sophora japonica* . In addition to well‐established clinical use in the management of chronic venous insufficiency and in improving capillary function, TROX has been shown to possess a broad‐spectrum of therapeutic properties [16]. These include antithrombotic, fibrinolytic, antioxidant, anti‐inflammatory, anti‐edema and neuroprotective activities [17, 18, 19]. Numerous studies have highlighted the antitumour potential of TROX in multiple cancer types, including colorectal, gastric, thyroid and liver cancers [13, 20, 21]. Notably, TROX displays an excellent safety profile, even in vulnerable populations such as elderly patients and pregnant women, where it has been well tolerated without significant adverse effects. Furthermore, TROX exhibits high oral bioavailability and the ability to cross the blood–brain barrier, making it a promising candidate for the treatment of central nervous system diseases [22]. Considering these findings, the present study aimed to evaluate the potential of TROX to enhance the antitumour efficacy of TMZ, the first‐line chemotherapy for the treatment of GBM, using an in vitro model based on human U87 GBM cells.

## 2 Materials and Methods

### 2.1 Cell Culture

The human GBM cell line U‐87 (U‐87 MG ATCC HTB‐14 *Homo sapiens* brain GBM IV grade) was used in this study and obtained from ATCC (American Type Culture Collection, Rockville, MD, USA). The cells were cultured in 75 cm^2^ flasks with Minimum Essential Medium Eagle (EMEM—Sigma‐Aldrich Catalogue No. M5650; St. Louis, MO, USA) supplemented with antibiotics (penicillin 1000 units—streptomycin 0.1 mg/L, Sigma‐Aldrich Catalogue No. P4333; St. Louis, MO, USA), L‐glutamine (GlutaMAX, ThermoFisher Scientific Catalogue No. 35050061; Waltham, MA, USA), Na pyruvate (Sodium pyruvate solution, Sigma‐Aldrich Catalogue No. S8636; St. Louis, MO, USA) and 10% (v/v) fetal bovine serum (FBS, Sigma‐Aldrich Catalogue No. 12103C; St. Louis, MO, USA) in a humidified environment with 5% CO_2_ and maintained at 37°C [23].

### 2.2 Cell Treatment

U‐87 cells were seeded in 96‐well plates at a density of 1 × 10^4^ cells per well, with a final volume of 150 μL. After 24 h, the cells were treated for an additional 24 h with TROX at concentrations of 10, 30, 100 or 300 μg/mL, TMZ 100 μM, and combinations of TMZ 100 μM with Troxerutin 10, 30, 100 or 300 μg/mL, all dissolved in the basal medium. The concentrations of TROX and TMZ were selected based on data obtained from our previous studies [21, 24, 25, 26].

### 2.3 Cell Viability Assay

The viability of U‐87 cells was assessed using the MTT assay, a mitochondria‐dependent colourimetric method. Cultures were pre‐treated with increasing concentrations of the test compound and incubated at 37°C with MTT (0.2 mg/mL) for 1 h. After incubation, the medium was removed and the cells were lysed using 100 μL of dimethyl sulfoxide. The reduction of MTT to formazan was quantified by measuring the optical density at 540 nm using a microplate reader.

### 2.4 Wound Healing Assay

The scratch assay was conducted to evaluate the effects of TMZ and its combination with TROX on U‐87 cell migration [27]. Briefly, U‐87 cells 2 × 10^6^ 8305C cells were plated on 60 mm plates (Corning Cell Culture, Tewksbury, MA, USA) in a volume of 2 mL. After 24 h, a scratch was made in the cell layer using a p200 pipette tip to create a straight‐line wound. The debris was then removed, and the medium was replaced with normal culture medium for the control group or supplemented with TMZ at 100 μM or combinations of TMZ 100 μM with TROX 30 or 100 μg/mL for 24 h. Then, cells were fixed with 4% paraformaldehyde (PFA) for 10 min and stained with 0.1% (w/v) crystal violet. The scratch area was quantified using ImageJ software (version 1.53a) to assess the extent of cell migration.

### 2.5 Colony Formation Assay

U‐87 cells were plated in six‐well plates at a density of 1000 cells per well and exposed to TMZ at 100 μM, combinations of TMZ 100 μM with TROX 30 and 100 μg/mL, or to the solvent alone as a control. After 24 h, the wells were washed with PBS and cultured in EMEM supplemented with antibiotics, L‐glutamine, Na pyruvate and 10% (v/v) fetal bovine. The cells were then incubated for 10 days, washed twice with PBS and stained with 0.1% (w/v) crystal violet [28].

### 2.6 Western Blot Analysis

After 24 h of treatment with basal medium for control (CTR) or TMZ 100 μM, combinations of TMZ 100 μM with TROX 30 and 100 μg/mL, GBM cells were washed twice with ice‐cold PBS, harvested and resuspended in a lysis buffer containing 20 mM Tris–HCl (pH 7.5), 10 mM NaF, 150 mM NaCl, 1% Nonidet P‐40 and a protease inhibitor cocktail (Roche, Monza, Italy). After 40 min, cell lysates were centrifuged at 16,000 *g* for 15 min at 4°C. Protein concentrations were measured using the Bio‐Rad protein assay with bovine serum albumin as a standard. The samples were then heated at 95°C for 5 min, and equal protein amounts were loaded onto an SDS‐PAGE gel and transferred to a PVDF (Immobilon‐P) membrane. Membranes were incubated overnight at 4°C with specific primary antibodies: Keap1 (1:500; Abcam, Cambridge, UK; AB150654), Nrf‐2 (1:500; Santa Cruz Biotechnology, Dallas, TX, USA; sc‐365949), HO‐1 (1:500; Santa Cruz Biotechnology, Dallas, TX, USA; sc‐136960). To detect multiple proteins, membranes were stripped and re‐probed. To ensure equal protein loading, blots were also incubated with a GAPDH antibody (1:500; Santa Cruz Biotechnology, Dallas, TX, USA; Sc‐32233). Protein bands were detected using the Advanced Chemiluminescence Detection System (ECL) reagent following the manufacturer's instructions (Thermo Fisher, Waltham, MA, USA). The densitometric analysis of protein expression was performed using BIORAD ChemiDoc XRS+ software, with GAPDH serving as an internal control for normalisation [29].

### 2.7 Terminal Deoxynucleotidyl Transferase dUTP Nick End Labeling (TUNEL) Assay

To assess the extent of GBM cell death, a TUNEL assay was performed using the “Fluorescein in Situ Cell Death Detection Kit” (Roche Diagnostics GmbH, Mannheim, Germany). After 24 h of treatment, cells were washed three times with PBS and incubated with a permeabilization solution containing 3% Triton X‐100 in PBS for 60 min at room temperature. Next, sections were incubated with terminal deoxynucleotidyl transferase (TdT) enzyme in a reaction buffer containing fluorescein‐dUTP for 90 min at 37°C. After labeling, the sections were washed in PBS and, for nuclear staining, 4′,6′‐diamidino‐2‐phenylindole (DAPI; Hoechst, Frankfurt, Germany) 2 μg/mL in PBS was added. To visualise the TUNEL‐positive cells, the sections were observed and photographed at 40× magnification using the Nikon Eclipse Ci‐L microscope.

### 2.8 Enzyme‐Linked Immunosorbent (ELISA) Assay Kit

IL‐1β, IL‐6, IL‐10, IL‐17, TNF‐α, ROMO1, GSH and SOD1 levels in the cell supernatant were evaluated according to the manufacturer's protocol (IL‐1 β ELISA Kit, RAB0273, Sigma‐Aldrich; IL‐10 ELISA Kit, KHC0101, Invitrogen; IL‐6 ELISA Kit, ab46027, Abcam; IL‐17 ELISA kit, SEA063Hu, Cloud‐Clone Corp.; TNF‐α ELISA kit, SEA133Hu, Cloud‐Clone Corp.; ROMO1 Elisa Kit, OKEH01371, Aviva; Glutathione ELISA kit, CEA294Ge, Cloud‐Clone Corp.; Cu/Zn Superoxide Dismutase ELISA Kit, QIA97, Sigma‐Aldrich).

### 2.9 Determination of Nitrite (${\mathrm{NO}}_{2}^{-}$) Levels

The nitrite production by U‐87 cells in response to the different treatments was analysed using the Griess colorimetric assay. U‐87 cells were cultured and exposed to the treatments. After 24 h, supernatants were collected to quantify NO₂^−^ levels. A total of 100 μL of each supernatant was placed in a microplate well, and an equal volume (100 μL) of Griess reagent was added. The reagent was prepared by combining 1% sulfanilamide and 0.1% N‐naphthylethyl‐ethylenediamine dihydrochloride in 5% phosphoric acid in a 1:1 ratio. The mixture was allowed to react at room temperature for 10 min, and absorbance was subsequently measured at 540 nm using a microplate reader. ${\mathrm{NO}}_{2}^{-}$ concentrations were calculated using a standard curve based on sodium nitrite [30].

### 2.10 Intracellular ROS

Intracellular ROS levels were quantified using dichloro‐dihydro‐fluorescein diacetate, DCFH‐DA (ROS Assay Kit—Fluorometric, AssayGenie, Dublin, Ireland, MAES0112). After treatment, cells were washed twice with PBS and incubated with 50 μM DCFH‐DA at 37°C for 40 min. Fluorescence intensity was measured using a microplate reader with excitation and emission wavelengths set at 485 and 530 nm, respectively.

### 2.11 Immunofluorescence Analysis

U‐87 cells were seeded on slides at a density of 2 × 10^5^ per 1 mL, and after 24 h, cells were exposed to the treatments. Following 24 h, cells were fixed on the slides with 4% PFA for 15 min and rinsed with PBS for 2 min. Permeabilization was performed using 0.2% Triton X‐100 in PBS, followed by blocking in 10% FBS in PBS for 10 min. The cells were then incubated overnight at 4°C with the appropriate primary antibodies: anti‐p53 (1:500; Santa Cruz Biotechnology, Dallas, TX, USA; sc‐126), anti‐E‐cadherin (1:500; Santa Cruz Biotechnology, Dallas, TX, USA; sc‐8526) and anti‐N‐cadherin (1:500; Santa Cruz Biotechnology, Dallas, TX, USA; sc‐8424). After washing with PBS, the slides were incubated with Alexa Fluor‐488‐conjugated secondary antibody (1:1000 v/v, Molecular Probes, Eugene, OR, USA) for 1 h at 37°C. For nuclear staining, DAPI (2 mg/mL in PBS; Hoechst, Frankfurt, Germany) was applied. Samples were imaged at 40× magnification using a Nikon Eclipse Ci‐L microscope [31].

### 2.12 Mitotracker Red Staining

U‐87 cells were cultured on glass slides, and after 24 h the treatments were added to the cells. The following day, MitoTracker Red CMXRos (Thermo Fisher Scientific, Cat. No. 7512) was applied for 45 min following the manufacturer's instructions. Cells were then washed with PBS and fixed in 4% PFA for 15 min. Fluorescence images were acquired using a Nikon Eclipse Ci‐L fluorescence microscope. The fluorescence intensity of the mitochondria relative to the cell volume was calculated using the ImageJ software.

### 2.13 DNA Fragmentation Assay

DNA fragmentation, indicative of apoptosis, was assessed in U‐87 cells via agarose gel electrophoresis. 1.5 × 10^6^ cells were seeded and exposed to the treatments for 24 h. Genomic DNA was subsequently isolated using the REDExtract‐N‐Amp Tissue PCR Kit (XNAT‐100RXN, Sigma‐Aldrich), following the manufacturer's protocol. Samples were subjected to electrophoresis on a 2% agarose gel at 90 V for 30 min. DNA bands were then visualised under UV illumination [32].

### 2.14 Determination of Antioxidant Activity Using the 2,2‐Diphenyl‐1‐Picrylhydrazyl (DPPH) Radical Scavenging Method

To determine the antioxidant activity of TROX, a 2,2‐Diphenyl‐1‐picrylhydrazyl (DPPH) radical scavenging method was performed. A methanolic DPPH solution (1 × 10^−4^ M) was prepared. Then, 1 mL aliquots of each aqueous sample were taken, including TROX at concentrations of 30 μg/mL or 100 μg/mL, TMZ at 100 μM and combinations of TMZ 100 μM with TROX 30 μg/mL or TROX 100 μg/mL. Two replicates were prepared for each sample, and 2 mL of the methanolic DPPH solution was added [33]. After being kept in the dark at room temperature for 1 h, the mixture's absorbance was measured at 517 nm with a microplate reader. The blank consisted of the methanolic DPPH solution. The radical scavenging activity was determined using the following formula:

$$\begin{array}{c} \text{Radical scavenging activity}\;\left(\%\right)\\ =\left\{\left({\mathrm{OD}}_{\text{control}}-{\mathrm{OD}}_{\text{sample}}\right)/{\mathrm{OD}}_{\text{control}}\right\}\times 100 \end{array}$$

### 2.15 Spheroid Cultures

To generate spheroids, 2% agarose solution was sterilised and melted in distilled water. 50 μL of the molten agarose, cooled to 40°C–45°C, was dispensed into each well of a 96‐well plate to form a thin layer and allowed to solidify at room temperature. Human GBM cell line U87 was grown to 70% confluence. Cells were seeded at a density of 5 × 10^5^ cells/mL in agar‐coated 96‐well plates. Plates were centrifuged at low speed (200 *g* for 15 min) to promote cell aggregation at the centre of the well. Subsequently, plates were incubated at 37°C in a humidified atmosphere with 5% CO_2_. Over 72 h, cells were induced to aggregate into a multicellular spheroid. Once complete spheroid formation was confirmed by light microscopy, cells were treated with TMZ at 100 μM and combinations of TMZ 100 μM with TROX 30 μg/mL or TROX 100 μg/mL for 24 h.

### 2.16 Spheroids Histology Analysis

Spheroid cultures were fixed in 4% PFA for 15 min and then stained with haematoxylin for 55 s and with eosin for 25 s. Sections were examined under a Nikon Eclipse Ci‐L optical microscope. The findings of histology are displayed at 2× (500 μm bar scale) magnification. The spheroid's size was measured using ImageJ software (version 1.53a).

### 2.17 Propidium Iodide (PI) Staining

Spheroids obtained from 3D cultures and treated with TMZ at 100 μM, and combinations of TMZ 100 μM with TROX 30 μg/mL or TROX 100 μg/mL were fixed with 4% PFA for 10 min. Then, they were washed three times with cold PBS at pH 7.4 and treated with propidium iodide (PI, 5 μg/mL, Sigma‐Aldrich China Inc., Shanghai, China) for dead cell staining. Afterward, the spheroids were washed again with PBS and transferred to microscope slides. A Nikon Eclipse Ci‐L optical microscope was used to detect fluorescence [34].

### 2.18 Immunofluorescence Analysis of Ki‐67 Expression in 3D Cell Cultures

After treatment with TMZ at 100 μM, and combinations of TMZ 100 μM with TROX 30 μg/mL or TROX 100 μg/mL, spheroids were fixed with 4% PFA for 30 min and then were washed with cold PBS. Permeabilization was performed using 0.2% Triton X‐100 in PBS, followed by blocking in 10% FBS in PBS for 1 h. The cells were then incubated overnight at 4°C with primary antibodies against Ki‐67 (1:500; Invitrogen; MA5‐14520). The nuclei were stained with DAPI. Afterward, the spheroids were transferred to microscope slides and observed by a Nikon Eclipse Ci‐L optical microscope.

## 3 Results

### 3.1 Synergistic Cytotoxic Effect of Troxerutin and Temozolomide in Human GBM Cells

**FIGURE 1:**
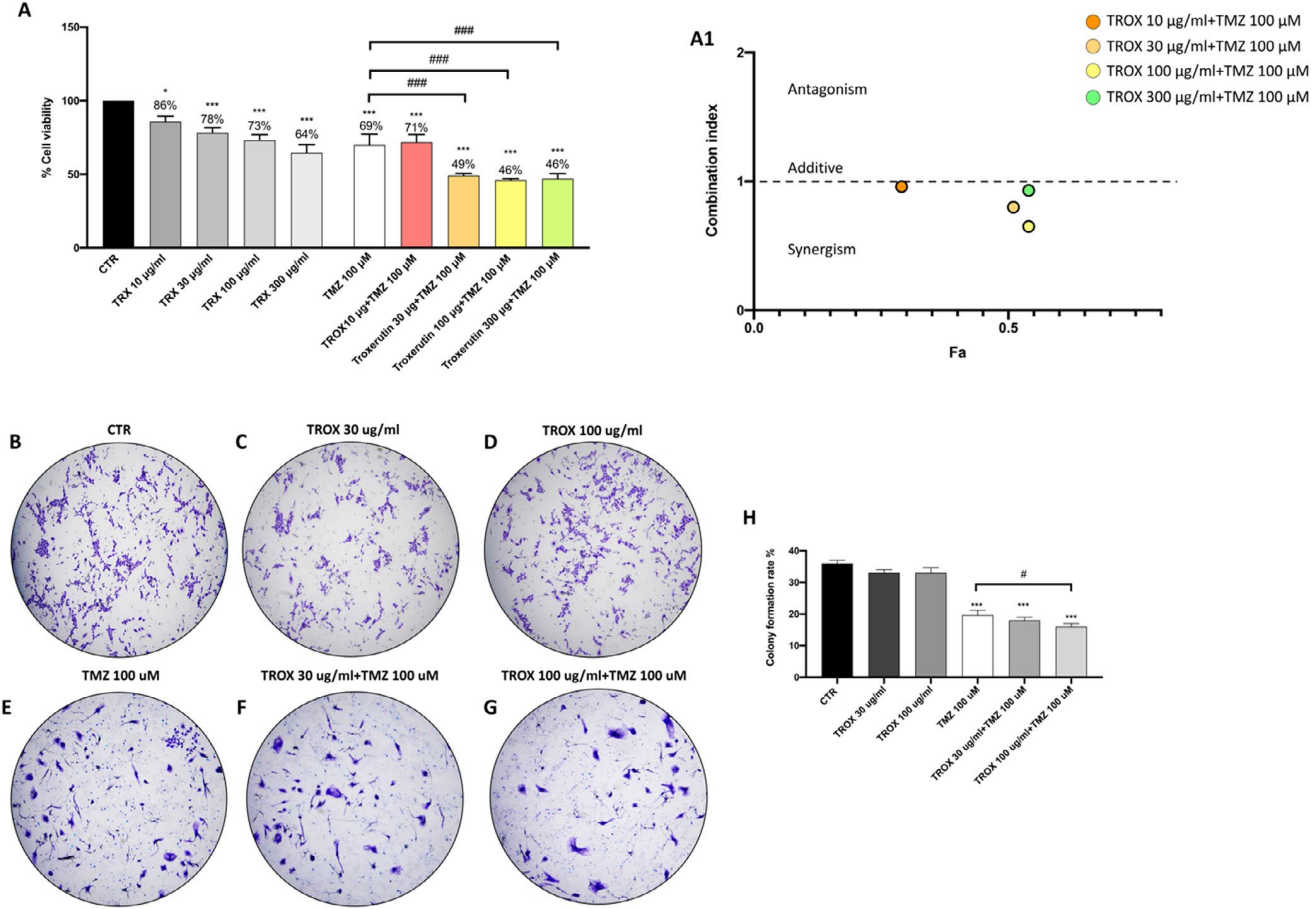
Effect of TROX, TMZ and their combination on cell viability and colony formation in U87 GBM cells. (A) Cell viability assay. (A1) CI analysis confirming synergism. (B) Colony formation assay after treatment with TROX at 30 and 100 μg/mL for 24 h did not show a significant reduction in colony‐forming ability compared to control (CTR) cells. (C) Colony formation assay after treatment with TROX 30 μg/ml for 24 h (D) Colony formation assay after treatment with TROX 100 μg/ml for 24 h. (E) Colony formation assay after treatment with TMZ 100 μM for 24 h (F‐G) Combined treatment with TROX (30 and 100 μg/ml respectively) and TMZ markedly suppressed colony formation, indicating a synergistic inhibition of cell growth (see scoring panel H). Data are representative of at least three independent experiments and are expressed as mean ± SD. Statistical analysis was performed using one‐way ANOVA followed by Bonferroni post hoc test. ***p < 0.001 versus CTR; #*p* < 0.05 versus TMZ 100 μM.

Cell viability was assessed using the MTT assay. As shown in Figure 1A, statistical analysis demonstrated that treatment with either TROX (30, 100 and 300 μg/mL) or TMZ (100 μM) alone appreciably decreased cell viability compared to untreated control cells. When combined, TROX at the lowest concentration tested (10 μg/mL) did not show significant differences compared with TMZ alone. In contrast, higher concentrations of TROX (30, 100 and 300 μg/mL) in combination with TMZ produced a significantly greater cytotoxic effect than TMZ alone. However, their combined administration produced a markedly enhanced cytotoxic effect. In support of this observation, the Combination Index (CI) calculated using CompuSyn was < 1, confirming a synergistic interaction between the two agents, as further illustrated in Figure 1 panel A1. Since the combination of 10 μg/mL TROX with TMZ did not result in a marked enhancement of cytotoxicity, we further investigated the clonogenic capacity of the cells using only the concentrations of TROX (30 and 100 μg/mL), both alone and in combination with TMZ. We observed that the combination of TROX 30 μg/mL (Figure 1C, score panel H) and TROX 100 μg/mL (Figure 1D, score panel H) did not significantly reduce the ability to form colonies compared to untreated control cells (Figure 1B, score panel H). Treatment with TMZ 100 μM (Figure 1E, score panel H) alone and in combination with TROX 30 μg/mL (Figure 1F, score panel H) and 100 μg/mL (Figure 1G, score panel H) significantly reduced the ability to form colonies compared to CTR cells; however, the combination of TMZ 100 μM and TROX 100 μM significantly improved the inhibition of colony formation compared to single treatment with TMZ 100 μM. This result indicates that TROX can potentiate TMZ‐induced cell growth inhibition in U87 cells. Based on these results, subsequent analyses were conducted focusing on the combination of TROX at 30 and 100 μg/mL with TMZ, in comparison to TMZ alone.

### 3.2 Combined Treatment With TROX and TMZ Impairs Cell Migration and Reverses EMT Marker Expression in GBM Cells

**FIGURE 2:**
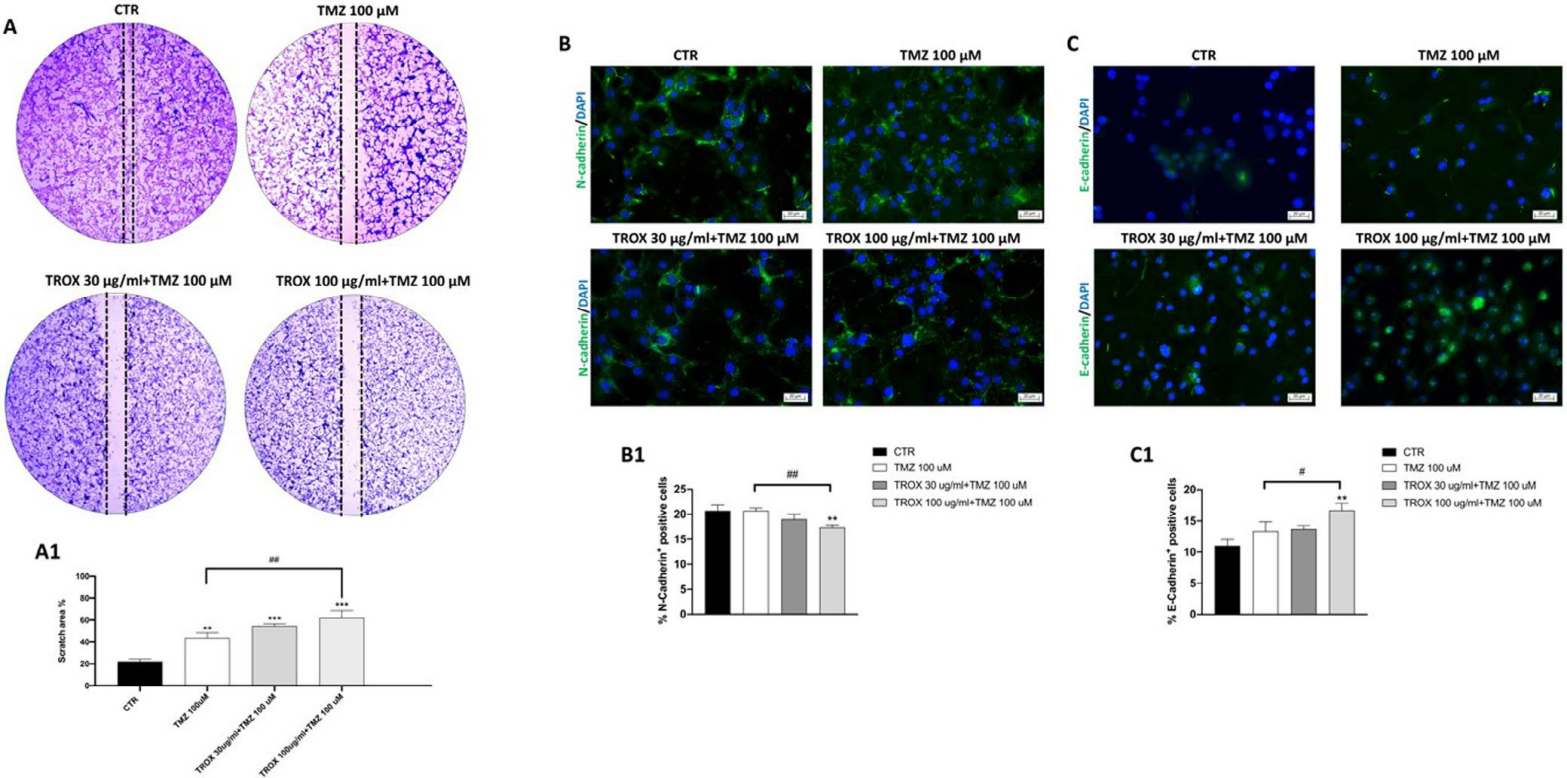
Effect of the co‐treatment of TROX and TMZ on cell migration and EMT markers in U87 cells. (A) Wound healing assay revealed a concentration‐dependent inhibition of cell migration and wound closure after 24 h of combined treatment with TMZ and TROX, compared to TMZ alone (see scoring panel A1). (B, B1) Immunofluorescence analysis of N‐Cadherin and (C, C1) E‐Cadherin. Both EMT markers showed that the combined treatment (100 μM TMZ + 100 μg/mL TROX) significantly decreased N‐cadherin expression and increased E‐cadherin expression, suggesting a shift toward a more epithelial phenotype. Data are representative of at least three independent experiments and are presented as mean ± SD. Statistical analysis was performed using one‐way ANOVA followed by Bonferroni post hoc test. *p < 0.05, **p < 0.01 vs. control (CTR); #*p* < 0.05, ##*p* < 0.01 versus TMZ 100 μM.

To evaluate the impact of TROX on cell migration, a wound‐healing assay was performed. As shown in Figure 2, treatment with TMZ alone reduced the migratory capacity of U87 cells. The combination of TMZ with TROX at 30 μg/mL further reduced the migratory capacity of U87 cells, whereas the combination with TROX at 100 μg/mL significantly enhanced this inhibitory effect compared to TMZ alone, as evidenced by the increase in scratch area after 24 h. To further investigate whether the reduced migratory capacity observed after TROX and TMZ treatment was associated with modulation of the epithelial–mesenchymal transition (EMT) programme, we analysed the expression of key EMT markers by immunofluorescence. The results demonstrated that only 24‐h treatment with TMZ 100 μM in combination with TROX 100 μg/mL resulted in a significant reduction in N‐cadherin, a mesenchymal marker (Figure 2B, scoring panel B1) and an increase in E‐cadherin, an epithelial marker (Figure 2C, scoring panel C1), suggesting an inhibition of the EMT process.

### 3.3 TROX Sensitises GBM Cells to TMZ‐Induced Apoptosis

**FIGURE 3:**
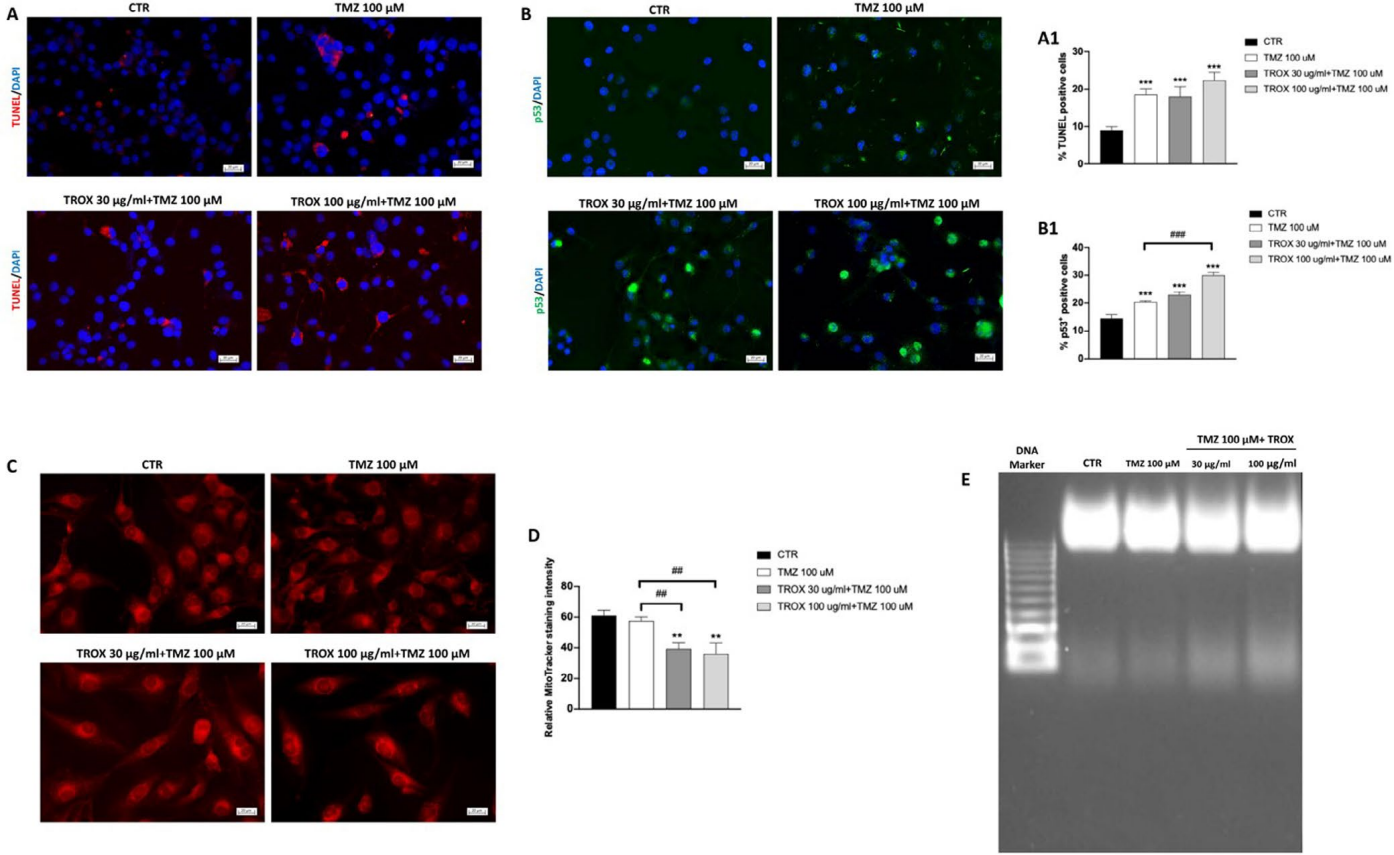
Apoptosis evaluation in GBM cells treated with the combination of TROX and TMZ. (A) TUNEL assay revealed low levels of apoptosis in untreated CTR cells. Treatment with 100 μM TMZ significantly increased the number of TUNEL‐positive nuclei. Co‐treatment of TROX (30 or 100 μg/mL) with TMZ further elevated the number of apoptotic cells in a concentration‐dependent manner. (B) Immunofluorescence staining of p53 showed a moderate increase in nuclear localization following TMZ treatment. Combination treatment with TROX resulted in a further increase in p53‐positive cells, with a statistically significant increase observed at 100 μg/mL TROX. (C) Mitochondrial membrane potential, assessed by MitoTracker Red staining, appeared disrupted in cells co‐treated with TMZ and TROX, consistent with mitochondrial dysfunction typically associated with apoptosis. (D) Quantification of MitoTracker Red fluorescence intensities per cell volume. (E) DNA fragmentation analysis revealed a characteristic apoptotic ladder pattern in cells treated with the combination of TMZ and TROX, more than that observed with TMZ treatment alone. Data are representative of at least three independent experiments. Values are means ± SDs. We used one‐way ANOVA tests followed by Bonferroni post hoc tests for multiple comparisons. ***p < 0.001 versus CTR; **p < 0.01 versus CTR; ## *p* < 0.01 versus TMZ 100 μM; ### *p* < 0.001 versus TMZ 100 μM.

To assess whether the combined treatment with TROX and TMZ induces apoptotic cell death in U87 cells, a TUNEL assay was performed (Figure 3A). The results showed minimal apoptosis in untreated control cells, whereas treatment with TMZ alone significantly increased TUNEL‐positive nuclei. Notably, combining TMZ with TROX at both 30 and 100 μg/mL further enhanced the number of apoptotic cells in a TROX concentration‐dependent manner, indicating a potentiation of TMZ‐induced apoptosis by TROX. To confirm the activation of apoptotic pathways, we analysed the expression of p53, a key regulator of apoptosis, by immunofluorescence analysis (Figure 3B). As expected, treatment with TMZ alone moderately increased nuclear p53 levels. However, the combined treatment with TROX resulted in increased nuclear accumulation of p53. Notably, a statistically significant increase in p53 expression was observed when TMZ was combined with 100 μg/mL TROX compared to TMZ alone, suggesting a potential activation of downstream apoptotic signalling pathways. Mitochondrial integrity was assessed using MitoTracker Red staining (Figure 3C). Furthermore, quantitative analysis of MitoTracker Red CMXRos fluorescence intensity confirmed a significant reduction in the co‐treated group, consistent with increased mitochondrial dysfunction and correlating with a higher level of apoptosis compared to TMZ alone and control cells (Figure 3D). Cells treated with the combination of TMZ and TROX showed a marked pattern of mitochondrial signalling intensity, consistent with mitochondrial dysfunction, which is a hallmark of apoptosis. Furthermore, DNA fragmentation analysis (Figure 3 panel E) confirmed these observations: the combined treatment induced a clear ladder‐like pattern, typical of apoptotic DNA cleavage, more evident than cells treated with TMZ alone.

### 3.4 TROX Enhances the Antioxidant Response in GBM Cells via Modulation of the KEAP1/NRF2 Pathway

**FIGURE 4:**
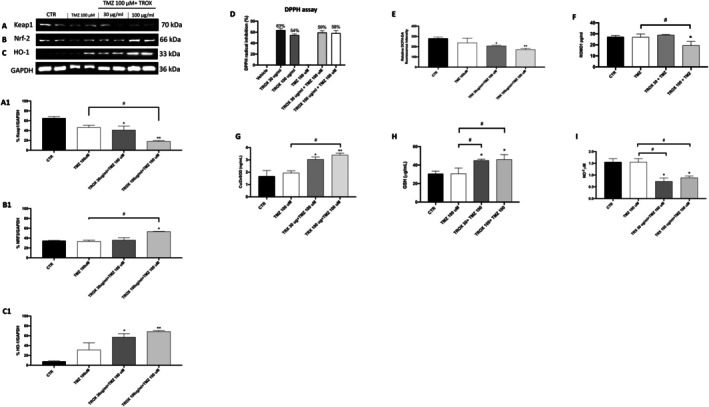
TROX in combination with TMZ modulated oxidative stress in U‐87 cells. The co‐administration of TROX and TMZ significantly modulated oxidative stress markers by altering the expression of (A, A1) Keap‐1, (B, B1) Nrf2 and (C, C1) HO‐1. (D) Free radical (DPPH) scavenging assay demonstrated a concentration‐dependent antioxidant activity of TROX. (E) Intracellular ROS quantification revealed a significant decrease in ROS levels upon combined treatment with TROX and TMZ. (F) ELISA analysis of ROMO1 protein levels indicated a marked reduction in cells treated with TROX (100 μg/mL) in combination with TMZ, compared to control and TMZ‐only groups. (G) ELISA tests of endogenous antioxidant defences Cu/Zn SOD and (H) GS. (I) Nitrite levels, assessed via the Griess assay, were significantly decreased in cells co‐treated with TMZ and TROX at both concentrations, compared to TMZ alone and untreated CTR cells. Data are representative of at least three independent experiments. Values are means ± SDs. We used one‐way ANOVA tests followed by Bonferroni post hoc tests for multiple comparisons. *p < 0.05 versus CTR; **p < 0.01 versus CTR; #*p* < 0.05 versus TMZ 100 μM.

TROX is known for its antioxidant properties, so we decided to further investigate its effect on the antioxidant pathway. Nrf2 is a major transcription factor involved in the regulation of oxidative stress and inflammation and plays a crucial role in GBM, contributing to tumour progression, tumour cell invasiveness and treatment resistance [35]. To test whether the combination of TROX and TMZ modulates the redox balance in GBM cells, we analysed the expression of key components of the KEAP1/NRF2 signalling pathway by Western blot. As shown in Figure 4, co‐treatment with TMZ and TROX at 30 and 100 μg/mL resulted in a marked reduction in the levels of the negative regulator of Nrf2, KEAP1 compared to control cells (Figure 4A, densitometric analysis panel A1). This effect was accompanied by a significant increase in NRF2 and HO‐1 expression after 24 h of TMZ treatment in association with TROX indicating the activation of the NRF2‐mediated antioxidant response (Figure 4B,C, densitometric analysis panel B1‐C1). To investigate the antioxidant potential of TROX, we performed a free radical (DPPH) scavenging assay. The results demonstrated that TROX possesses a strong antioxidant activity in a concentration‐dependent manner, supporting its ability to counteract oxidative stress (Figure 4D). In agreement with these results, the analysis of intracellular ROS levels showed that the combination of TMZ with troxerutin resulted in a significant reduction of cellular oxidative stress (Figure 4E). ROMO1, a mitochondrial protein involved in ROS regulation, is known to be overexpressed in several neoplastic cells and implicated in tumour invasion and progression. In support of the observed antioxidant effect, ELISA assays of ROMO1 showed a marked decrease in protein levels in cells treated with TMZ in combination with TROX (100 μg/mL), compared to both CTR cells and cells treated with TMZ alone (Figure 4F). In addition, ELISA tests for intracellular antioxidant defences revealed that co‐treatment of TMZ with TROX (both at 30 and 100 μg/mL) significantly increased GSH and SOD1 levels compared to both TMZ alone and untreated cells, further confirming the antioxidant properties of TROX (Figure 4G,H). Finally, the Griess assay performed on the cell supernatant demonstrated that the combined treatment of TMZ with TROX at both doses of 30 and 100 μg/mL significantly reduced nitrite levels compared to CTR cells and cells treated with TMZ alone (Figure 4I).

### 3.5 Anti‐Inflammatory Effects of the TMZ and TROX Combination Treatment

**FIGURE 5:**
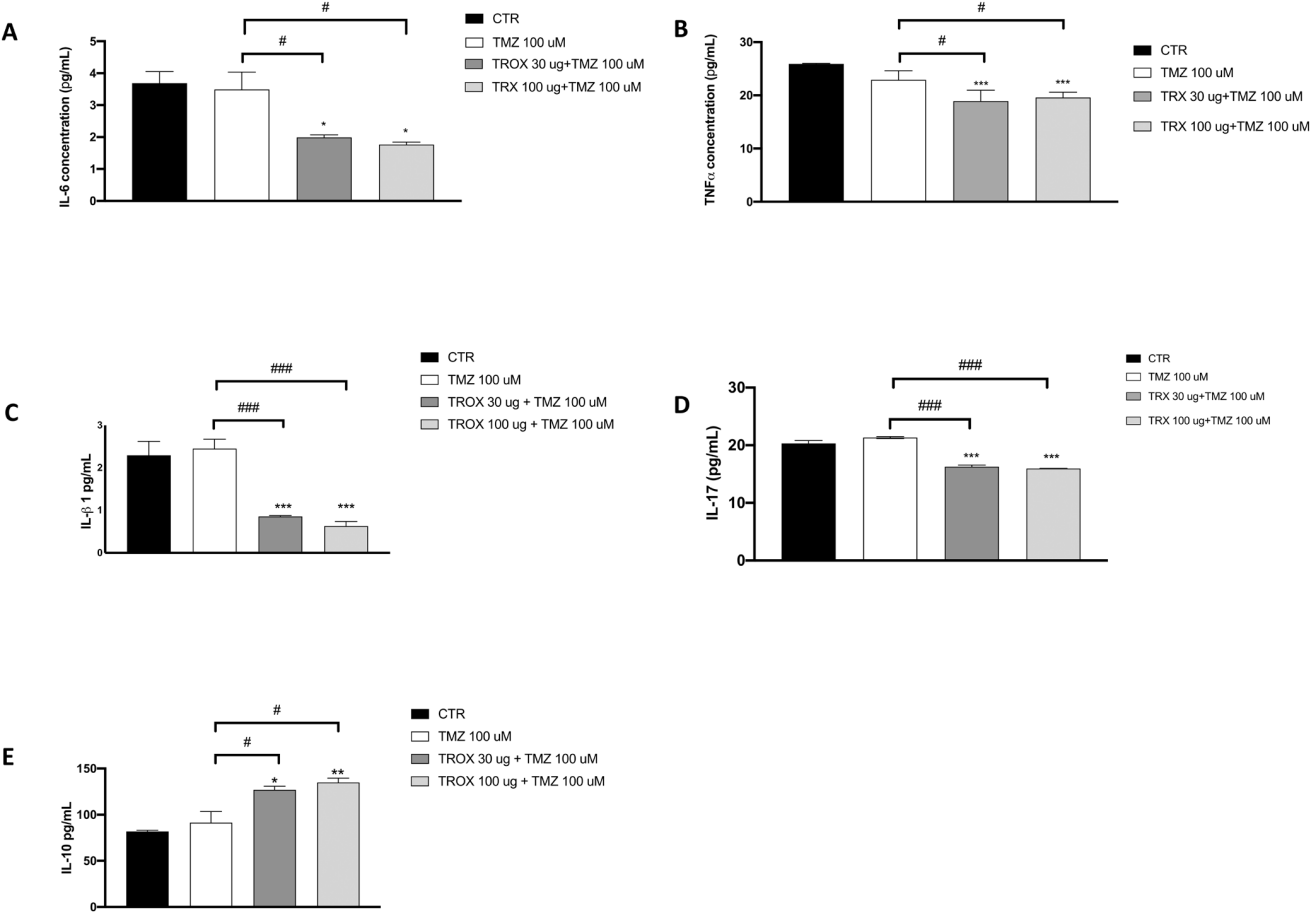
Effect of the combination of TROX and TMZ on inflammatory cytokine. ELISA assay revealed a significant reduction in the levels of the pro‐inflammatory cytokines (A) IL‐6, (B) TNF‐α, (C) IL‐1β, (D) IL‐17. (E) Conversely, a marked increase was observed in the anti‐inflammatory cytokines IL‐10. Data are representative of at least three independent experiments. Values are means ± SDs. We used one‐way ANOVA tests followed by Bonferroni post hoc tests for multiple comparisons. *p < 0.05 versus CTR; **p < 0.01 versus CTR; ***p < 0.001 versus CTR; #*p* < 0.05 versus TMZ 100 μM; ###*p* < 0.001 versus TMZ 100 μM.

Altered ROS levels are known to promote the release of pro‐inflammatory cytokines, contributing to the development of chronic inflammation that promotes chemoresistance, cell survival, angiogenesis, metastasis and tumour progression [36]. In light of the well‐documented anti‐inflammatory activity of TROX, we investigated its potential inhibitory effects on the production of inflammatory cytokines, focusing in particular on IL‐6, TNF‐α and IL‐1β. ELISA assays showed that the combination of TMZ with troxerutin significantly reduced the levels of the pro‐inflammatory cytokines IL‐6 (Figure 5A), TNF‐α (Figure 5B) and IL‐1β (Figure 5C), whereas it significantly increased the levels of the anti‐inflammatory cytokines IL‐17 (Figure 5D) and IL‐10 (Figure 5E).

### 3.6 Combination of TMZ and TROX Reduces Spheroid Growth and Proliferation in U87 Cells

**FIGURE 6:**
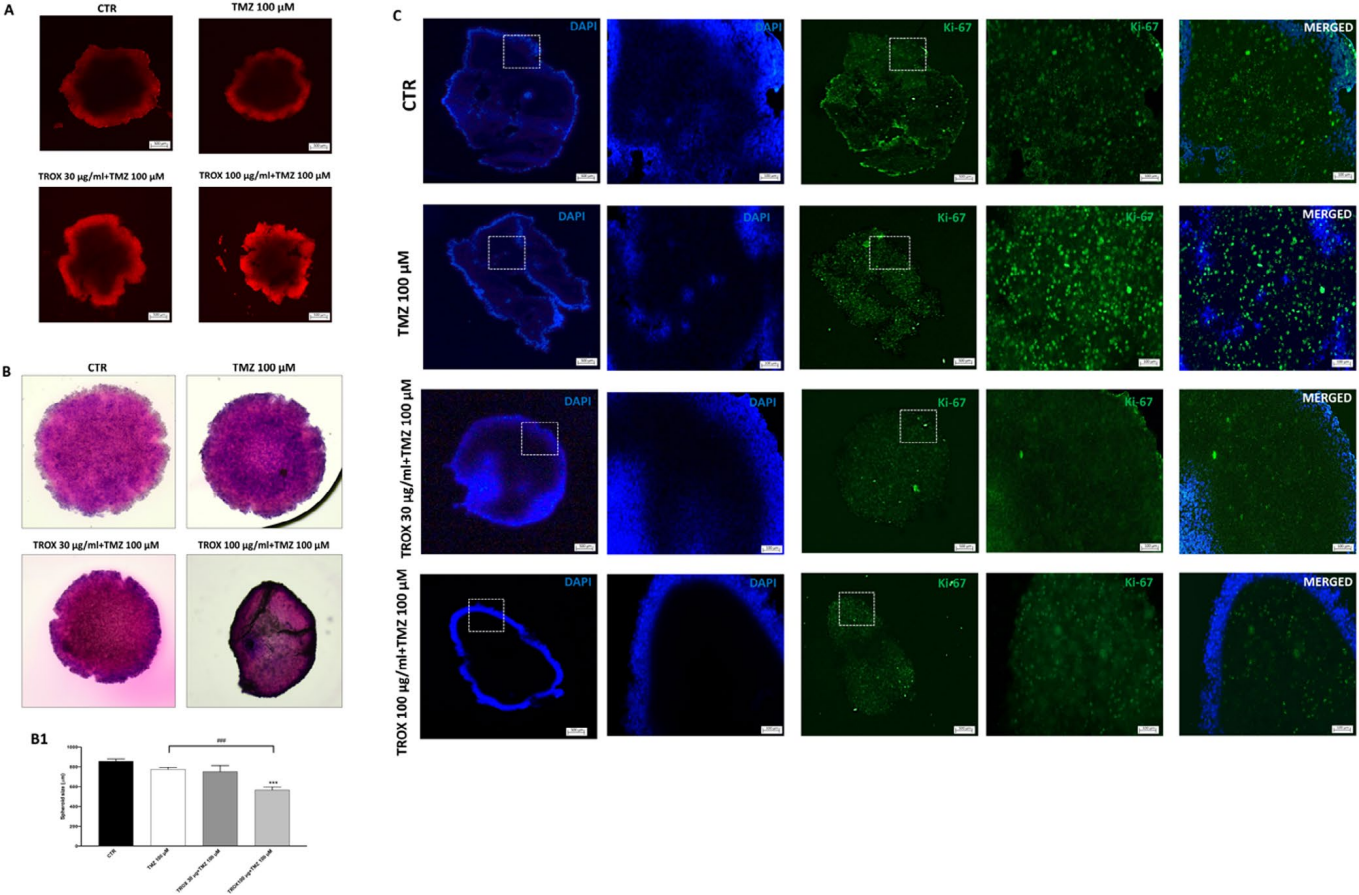
TMZ combined with TROX enhanced the inhibition of U87 spheroid growth and proliferation compared to TMZ alone. (A) Propidium iodide staining revealed significantly increased fluorescence intensity in spheroids exposed to TMZ + TROX (100 μg/mL) in comparison to untreated CTR and TMZ cells. (B, B1) Haematoxylin and eosin staining demonstrated a marked decrease in the spheroid area following combined treatment with TMZ (100 μM) and TROX (100 μg/mL). (C) Immunofluorescence analysis of the proliferation marker Ki‐67 revealed a substantial reduction in staining intensity in spheroids treated with TMZ + TROX at both 30 μg/mL and 100 μg/mL concentrations, compared to CTR and TMZ alone. *p < 0.05 versus CTR; ***p < 0.001 versus CTR; ###*p* < 0.001 versus TMZ 100 μM.

To evaluate the efficacy of the TMZ and TROX combination in a three‐dimensional glioma model, U87‐derived spheroids were generated and analysed using multiple staining methods. Propidium iodide (PI) staining revealed a markedly higher fluorescence intensity in spheroids treated with TMZ + TROX (100 μg/mL), indicating an increase in cell death compared to both untreated CTR and TMZ alone (Figure 6A). Haematoxylin–eosin (H&E) staining further demonstrated a significant reduction in spheroid size in the combination group (TROX 100 μg/mL + TMZ 100 μM), supporting a pronounced anti‐growth effect (Figure 6B, score B1). Additionally, immunofluorescence analysis for Ki‐67 staining revealed decreased proliferative activity in both TMZ + TROX 30 μg/mL and TMZ + TROX 100 μg/mL groups, as evidenced by a lower fluorescence signal compared to control and TMZ‐treated spheroids (Figure 6C). Collectively, these results indicate that TROX enhances the antitumour activity of TMZ in the 3D GBM model by promoting cell death and suppressing both tumour growth and proliferation.

## 4 Discussion

The present study demonstrated that TROX, a natural flavonoid with well‐established antioxidant and anti‐inflammatory properties, can significantly enhance the antitumor effects of TMZ in human GBM U87 cells, paving the way for the potential application of TROX as an adjuvant agent to improve the efficacy and tolerability of standard chemotherapy in the treatment of GBM. Natural flavonoids like TROX are emerging as a new class of anticancer agents with minimal side effects [13, 20, 21, 37]; however, their potential in GBM and their combination with TMZ are still poorly investigated. In this context, combinatorial strategies aimed at sensitising tumour cells to TMZ‐induced cytotoxicity while mitigating its adverse effects are highly desirable [38, 39]. TROX significantly potentiates the cytotoxic and antiproliferative effects of TMZ, suggesting a synergistic interaction. Cell migration and EMT are critical processes in GBM invasiveness and progression [40]. Our wound healing assay confirmed that the combination of TMZ and TROX significantly inhibited GBM cell migration compared to TMZ alone. Furthermore, immunofluorescent analysis of EMT markers revealed a partial reversal of the mesenchymal phenotype, characterised by increased E‐cadherin expression and decreased N‐cadherin. These results suggest that TROX may contribute to the suppression of GBM aggressiveness by interfering with the dynamics of EMT. TMZ is widely recognised for its ability to induce apoptosis in tumour cells [41]; however, its efficacy is often compromised by severe side effects that force many patients to discontinue treatment. It is essential to identify compounds able to sensitise tumour cells to TMZ‐induced cell death, allowing the achievement of an effective therapeutic response even at lower doses and with less toxicity to healthy cells. Our data showed that TROX significantly enhances TMZ‐induced apoptosis in U87 cells. This effect was confirmed by TUNEL assay, mitochondrial dysfunction analysis and DNA fragmentation assessment, indicating an increased activation of apoptotic pathways. The combined treatment resulted in a nuclear accumulation of p53, a key pro‐apoptotic marker, strengthening the hypothesis that TROX potentiates the pro‐apoptotic effect of TMZ. These findings are consistent with previous evidence showing that TROX enhanced 5‐FU‐induced apoptosis in gastric cancer cells, suggesting its potential as a broad‐spectrum adjuvant to enhance chemotherapy efficacy via apoptotic signalling [37]. In GBM, cellular metabolism is profoundly altered, with intense metabolic activity and basal oxidative stress levels. Oxidative stress develops due to a disproportion between the synthesis and accumulation of free radicals like ROS and RNS [42]. Moreover, brain tissue demands significantly more oxygen and energy compared to other organs to sustain normal physiological functions. Its high content of polyunsaturated fatty acids and low levels of endogenous antioxidants make it especially prone to oxidative stress [43]. This state of oxidative stress actively contributes to tumour progression, as ROS act as crucial mediators in signal transduction processes, promoting cell proliferation and hindering apoptosis [44]. Our data demonstrated that TROX activated the KEAP1/NRF2/HO‐1 axis, enhancing antioxidant defence. Similarly, Nisha Susan et al. demonstrated that TROX modulates the NRF2 pathway in hepatocellular carcinoma, reinforcing its role as a redox‐modulating agent with potential applications across different cancer types [13]. Studies have reported that NRF2 suppresses tumorigenesis via transcriptionally activating the cytoprotective genes NAD(P)H Quinone Oxidoreductase I (NQO1) and HO‐1 [45]. NRF2 interacts with cytokines such as TNF‐α and IL‐6, linking oxidative stress to inflammation and tumorigenesis. Our results show that the TROX–TMZ combination reduces pro‐inflammatory cytokines (IL‐6, TNF‐α, IL‐1β) and increases IL‐10, highlighting its anti‐inflammatory potential. Moreover, by restoring levels of antioxidants like SOD, CAT and GSH—often depleted in GBM—this treatment may counteract oxidative stress and reduce tumour aggressiveness [10, 46]. The observed reduction in intracellular ROS, nitrite levels and ROMO1 expression, along with the increased levels of antioxidant defences such as GSH and SOD1, supports the antioxidant potential of TROX in GBM cells. To further validate these findings, 3D spheroid models were employed, showing that the combination of TROX and TMZ significantly inhibited growth in a more physiologically relevant tumour‐like context, thereby reinforcing its promise as an effective adjuvant strategy in GBM therapy. In summary, our results demonstrate that TROX, thanks to its antioxidant and anti‐inflammatory properties, can significantly enhance the antitumor efficacy of TMZ in human GBM U87 cells. The observed synergistic effect is evidenced by increased apoptosis, reduced cell migration and EMT, as well as modulation of oxidative stress and inflammation. Activation of the KEAP1/NRF2 axis and regulation of inflammatory cytokines suggest that TROX may act at multiple levels, interfering with mechanisms that contribute to tumour progression and chemoresistance.

## 5 Conclusions

This study provides comprehensive evidence that TROX, a natural flavonoid with established antioxidant and anti‐inflammatory properties, can significantly potentiate the antitumor effects of TMZ in human GBM U87 cells. The combined treatment induced an enhanced reduction in cell viability and clonogenic potential, enhanced apoptotic cell death via p53 nuclear accumulation, mitochondrial dysfunction and DNA fragmentation, and suppressed migratory capacity by reversing EMT markers. Furthermore, the combination modulated oxidative stress by activating the KEAP1/NRF2/HO‐1 axis, restoring intracellular antioxidant defences such as GSH and SOD1, and reducing pro‐inflammatory cytokines including IL‐6, TNF‐α and IL‐1β, whereas increasing anti‐inflammatory mediators such as IL‐10 and IL‐17. These effects were confirmed in 3D spheroid models, demonstrating the translational relevance of the findings in a physiologically relevant tumour microenvironment. Overall, our results demonstrate that TROX can potentiate TMZ efficacy at multiple cellular and molecular levels, targeting proliferation, apoptosis, oxidative stress and inflammatory pathways simultaneously. This multifaceted adjuvant effect suggests that TROX has the potential to improve therapeutic outcomes, mitigate chemoresistance and reduce treatment‐associated toxicity in GBM. Further in vivo studies and mechanistic investigations are warranted to fully elucidate the molecular interactions underlying the observed effects and to assess the safety and efficacy of this combination in preclinical models.

## Author Contributions

**Deborah Mannino:** data curation (equal), validation (equal), writing – original draft (equal). **Elsa Calcaterra:** data curation (equal), methodology (equal), writing – original draft (equal). **Sarah Adriana Scuderi:** data curation (equal), methodology (equal). **Maria Caffo:** data curation (equal), methodology (equal). **Fabiola De Luca:** data curation (equal), methodology (equal). **Antonio Catalfamo:** data curation (equal), methodology (equal). **Giovanna Casili:** conceptualization (equal), supervision (equal), validation (equal). **Irene Paterniti:** conceptualization (equal), supervision (equal), validation (equal).

## Conflicts of Interest

The authors declare no conflicts of interest.

## Data Availability

The data that support the findings of this study are available from the corresponding author upon reasonable request.

## References

[1] M. A. Horowitz , A. Ghadiyaram , Y. Mehkri , et al., “Surgical Resection of Glioblastoma in the Very Elderly: An Analysis of Survival Outcomes Using the Surveillance, Epidemiology, and End Results Database,” Clinical Neurology and Neurosurgery 245 (2024): 108469, 10.1016/j.clineuro.2024.108469. 39079287

[2] M. Jezierzanski , N. Nafalska , M. Stopyra , et al., “Temozolomide (TMZ) in the Treatment of Glioblastoma Multiforme‐A Literature Review and Clinical Outcomes,” Current Oncology 31 (2024): 3994–4002, 10.3390/curroncol31070296. 39057168 PMC11275351

[3] S. T. Asma , U. Acaroz , K. Imre , et al., “Natural Products/Bioactive Compounds as a Source of Anticancer Drugs,” Cancers (Basel) 14 (2022): 14, 10.3390/cancers14246203. PMC9777303 36551687

[4] A. Majchrzak‐Celinska and E. Studzinska‐Sroka , “New Avenues and Major Achievements in Phytocompounds Research for Glioblastoma Therapy,” Molecules 29 (2024): 1682, 10.3390/molecules29071682. 38611962 PMC11013944

[5] C. Rodriguez‐Garcia , C. Sanchez‐Quesada , and J. J. Gaforio , “Dietary Flavonoids as Cancer Chemopreventive Agents: An Updated Review of Human Studies,” Antioxidants (Basel) 8 (2019): 137, 10.3390/antiox8050137. 31109072 PMC6562590

[6] J. Hou , Z. Xing , A. Li , et al., “Synergistic Antitumor Effects of Phlorizin and Temozolomide in Glioblastoma: Mechanistic Insights and Molecular Targeting,” Fitoterapia 180 (2025): 106313, 10.1016/j.fitote.2024.106313. 39617291

[7] G. Ke , P. Hu , H. Xiong , et al., “Enhancing Temozolomide Efficacy in GBM: The Synergistic Role of Chuanxiong Rhizoma Essential Oil,” Phytomedicine 140 (2025): 156575, 10.1016/j.phymed.2025.156575. 40088740

[8] O. Canli , A. M. Nicolas , J. Gupta , et al., “Myeloid Cell‐Derived Reactive Oxygen Species Induce Epithelial Mutagenesis,” Cancer Cell 32 (2017): 869–883, 10.1016/j.ccell.2017.11.004. 29232557

[9] A. Arabzadeh , T. Mortezazadeh , T. Aryafar , E. Gharepapagh , M. Majdaeen , and B. Farhood , “Therapeutic Potentials of Resveratrol in Combination With Radiotherapy and Chemotherapy During Glioblastoma Treatment: A Mechanistic Review,” Cancer Cell International 21 (2021): 391, 10.1186/s12935-021-02099-0. 34289841 PMC8296583

[10] M. J. Ramirez‐Exposito and J. M. Martinez‐Martos , “The Delicate Equilibrium Between Oxidants and Antioxidants in Brain Glioma,” Current Neuropharmacology 17 (2019): 342–351, 10.2174/1570159X16666180302120925. 29512467 PMC6482474

[11] J. A. Campos‐Sandoval , M. C. Gomez‐Garcia , J. L. Santos‐Jimenez , J. M. Mates , F. J. Alonso , and J. Marquez , “Antioxidant Responses Related to Temozolomide Resistance in Glioblastoma,” Neurochemistry International 149 (2021): 105136, 10.1016/j.neuint.2021.105136. 34274381

[12] K. A. Conklin , “Dietary Antioxidants During Cancer Chemotherapy: Impact on Chemotherapeutic Effectiveness and Development of Side Effects,” Nutrition and Cancer 37 (2000): 1–18, 10.1207/S15327914NC3701_1. 10965514

[13] N. S. Thomas , K. George , and A. A. A. Selvam , “Anticancer Mechanism of Troxerutin via Targeting Nrf2 and NF‐kappaB Signalling Pathways in Hepatocarcinoma Cell Line,” Toxicology In Vitro 54 (2019): 317–329, 10.1016/j.tiv.2018.10.018. 30389603

[14] P. Elangovan , A. M. Jalaludeen , R. Ramakrishnan , K. Amutha , and L. Pari , “In‐Vivo and In‐Vitro Antioxidant Activity of Troxerutin on Nickel Induced Toxicity in Experimental Rats,” Iranian Journal of Pharmaceutical Research 19 (2020): 89–97, 10.22037/ijpr.2020.15487.13126. 33224214 PMC7667551

[15] A. Z. Darkazally , A. Alnour , and S. Homsi , “Troxerutin Effect on Gastric Ulcers Induced by Ketorolac in Rats: Relation With Oxidative Stress,” Heliyon 10 (2024): e38893, 10.1016/j.heliyon.2024.e38893. 39435065 PMC11492593

[16] C. B. Miguel , R. S. Andrade , L. Mazurek , et al., “Emerging Pharmacological Interventions for Chronic Venous Insufficiency: A Comprehensive Systematic Review and Meta‐Analysis of Efficacy, Safety, and Therapeutic Advances,” Pharmaceutics 17 (2025): 59, 10.3390/pharmaceutics17010059. 39861707 PMC11768779

[17] A. A. Sowunmi , N. A. Omeiza , A. Bakre , H. A. Abdulrahim , and A. O. Aderibigbe , “Dissecting the Antidepressant Effect of Troxerutin: Modulation of Neuroinflammatory and Oxidative Stress Biomarkers in Lipopolysaccharide‐Treated Mice,” Naunyn‐Schmiedeberg's Archives of Pharmacology 397 (2024): 9965–9979, 10.1007/s00210-024-03252-y. 38951153

[18] Y. Yu and G. Zheng , “Troxerutin Protects Against Diabetic Cardiomyopathy Through NF‐kappaB/AKT/IRS1 in a Rat Model of Type 2 Diabetes,” Molecular Medicine Reports 15 (2017): 3473–3478, 10.3892/mmr.2017.6456. 28440404 PMC5436284

[19] X. Ping , J. Junqing , J. Junfeng , and J. Enjin , “Radioprotective Effects of Troxerutin Against Gamma Irradiation in Mice Liver,” International Journal of Radiation Biology 88 (2012): 607–612, 10.3109/09553002.2012.692494. 22571496

[20] J. Yu , X. Huang , M. Cao , et al., “Anticancer Effect of Troxerutin in Human Non‐Small‐Cell Lung Cancer Cell A549 and Inhibition of Tumor Formation in BALB/c Nude Mice,” Journal of Environmental Pathology, Toxicology and Oncology 40 (2021): 25–35, 10.1615/JEnvironPatholToxicolOncol.2021037951. 34587402

[21] V. Bova , R. Basilotta , G. Casili , et al., “The Protective Role of Troxerutin (Trox) in Counteracting Anaplastic Thyroid Carcinoma (ATC) Progression,” Biomedicine 12 (2024): 1755, 10.3390/biomedicines12081755. PMC11351865 39200219

[22] F. Liu , Y. Xu , L. Rui , S. Gao , H. Dong , and Q. Guo , “Liquid Chromatography/Tandem Mass Spectrometry Assay for the Quantification of Troxerutin in Human Plasma,” Rapid Communications in Mass Spectrometry 20 (2006): 3522–3526, 10.1002/rcm.2764. 17072901

[23] A. Filippone , G. Casili , S. A. Scuderi , et al., “Sodium Propionate Contributes to Tumor Cell Growth Inhibition Through PPAR‐Gamma Signaling,” Cancers 15 (2022): 217, 10.3390/cancers15010217. 36612214 PMC9818202

[24] M. Campolo , M. Lanza , G. Casili , et al., “TAK1 Inhibitor Enhances the Therapeutic Treatment for Glioblastoma,” Cancers (Basel) 13 (2020): 41, 10.3390/cancers13010041. 33375627 PMC7794959

[25] F. Atif , N. R. Patel , S. Yousuf , and D. G. Stein , “The Synergistic Effect of Combination Progesterone and Temozolomide on Human Glioblastoma Cells,” PLoS One 10 (2015): e0131441, 10.1371/journal.pone.0131441. 26110872 PMC4482510

[26] M. Pazhouhi , R. Sariri , M. R. Khazaei , M. T. Moradi , and M. Khazaei , “Synergistic Effect of Temozolomide and Thymoquinone on Human Glioblastoma Multiforme Cell Line (U87MG),” Journal of Cancer Research and Therapeutics 14 (2018): 1023–1028, 10.4103/0973-1482.187241. 30197342

[27] C. C. Liang , A. Y. Park , and J. L. Guan , “In Vitro Scratch Assay: A Convenient and Inexpensive Method for Analysis of Cell Migration In Vitro,” Nature Protocols 2 (2007): 329–333, 10.1038/nprot.2007.30. 17406593

[28] R. Basilotta , G. Casili , D. Mannino , et al., “Benzyl Isothiocyanate Suppresses Development of Thyroid Carcinoma by Regulating Both Autophagy and Apoptosis Pathway,” Iscience 27 (2024): 110796, 10.1016/j.isci.2024.110796. 39398237 PMC11471196

[29] V. Bova , D. Mannino , A. E. Salako , E. Esposito , A. Filippone , and S. A. Scuderi , “Casein Kinase 2 Inhibitor, CX‐4945, Induces Apoptosis and Restores Blood‐Brain Barrier Homeostasis in In Vitro and In Vivo Models of Glioblastoma,” Cancers 16 (2024): 3936, 10.3390/cancers16233936. 39682125 PMC11640555

[30] F. De Gaetano , D. Mannino , C. Celesti , et al., “Randomly Methylated β‐Cyclodextrin Improves Water—Solubility, Cellular Protection and Mucosa Permeability of Idebenone,” International Journal of Pharmaceutics 665 (2024): 124718, 10.1016/j.ijpharm.2024.124718. 39288841

[31] S. A. Scuderi , G. Casili , A. Ardizzone , et al., “KYP‐2047, an Inhibitor of Prolyl‐Oligopeptidase, Reduces GlioBlastoma Proliferation Through Angiogenesis and Apoptosis Modulation,” Cancers 13 (2021): 3444, 10.3390/cancers13143444. 34298658 PMC8306782

[32] K. Ohnishi , I. Ota , K. Yane , et al., “Glycerol as a Chemical Chaperone Enhances Radiation‐Induced Apoptosis in Anaplastic Thyroid Carcinoma Cells,” Molecular Cancer 1 (2002): 4, 10.1186/1476-4598-1-4. 12423550 PMC140146

[33] N. Chaves , A. Santiago , and J. C. Alias , “Quantification of the Antioxidant Activity of Plant Extracts: Analysis of Sensitivity and Hierarchization Based on the Method Used,” Antioxidants (Basel) 9 (2020): 76, 10.3390/antiox9010076. 31952329 PMC7023273

[34] V. M. Le , M. D. Lang , W. B. Shi , and J. W. Liu , “A Collagen‐Based Multicellular Tumor Spheroid Model for Evaluation of the Efficiency of Nanoparticle Drug Delivery,” Artificial Cells, Nanomedicine, and Biotechnology 44 (2016): 540–544, 10.3109/21691401.2014.968820. 25315504

[35] W. A. Awuah , A. R. Toufik , R. Yarlagadda , et al., “Exploring the Role of Nrf2 Signaling in Glioblastoma Multiforme,” Discover Oncology 13 (2022): 94, 10.1007/s12672-022-00556-4. 36169772 PMC9519816

[36] A. Salazar‐Ramiro , D. Ramirez‐Ortega , V. Perez de la Cruz , et al., “Role of Redox Status in Development of Glioblastoma,” Frontiers in Immunology 7 (2016): 156, 10.3389/fimmu.2016.00156. 27199982 PMC4844613

[37] G. Y. Xu and X. J. Tang , “Troxerutin (TXN) Potentiated 5‐Fluorouracil (5‐Fu) Treatment of Human Gastric Cancer Through Suppressing STAT3/NF‐kappaB and Bcl‐2 Signaling Pathways,” Biomedicine & Pharmacotherapy 92 (2017): 95–107, 10.1016/j.biopha.2017.04.059. 28531805

[38] S. H. Tai , Y. W. Lin , T. Y. Huang , et al., “Cinnamophilin Enhances Temozolomide‐Induced Cytotoxicity Against Malignant Glioma: The Roles of ROS and Cell Cycle Arrest,” Translational Cancer Research 10 (2021): 3906–3920, 10.21037/tcr-20-3426. 35116690 PMC8798401

[39] Z. Chen , G. Zhu , C. Sheng , J. Lei , S. Song , and J. Zhu , “Hispidulin Enhances Temozolomide (TMZ)‐Induced Cytotoxicity Against Malignant Glioma Cells In Vitro by Inhibiting Autophagy,” Computational Intelligence and Neuroscience 2022 (2022): 5266770, 10.1155/2022/5266770. 35800695 PMC9256375

[40] Y. Iwadate , “Epithelial‐Mesenchymal Transition in Glioblastoma Progression,” Oncology Letters 11 (2016): 1615–1620, 10.3892/ol.2016.4113. 26998052 PMC4774466

[41] M. T. Tomicic , R. Meise , D. Aasland , et al., “Apoptosis Induced by Temozolomide and Nimustine in Glioblastoma Cells Is Supported by JNK/c‐Jun‐Mediated Induction of the BH3‐Only Protein BIM,” Oncotarget 6 (2015): 33755–33768, 10.18632/oncotarget.5274. 26418950 PMC4741800

[42] M. Rinaldi , M. Caffo , L. Minutoli , et al., “ROS and Brain Gliomas: An Overview of Potential and Innovative Therapeutic Strategies,” International Journal of Molecular Sciences 17 (2016): 984, 10.3390/ijms17060984. 27338365 PMC4926513

[43] M. E. Watts , R. Pocock , and C. Claudianos , “Brain Energy and Oxygen Metabolism: Emerging Role in Normal Function and Disease,” Frontiers in Molecular Neuroscience 11 (2018): 216, 10.3389/fnmol.2018.00216. 29988368 PMC6023993

[44] X. Qi , S. K. Jha , N. K. Jha , et al., “Antioxidants in Brain Tumors: Current Therapeutic Significance and Future Prospects,” Molecular Cancer 21 (2022): 204, 10.1186/s12943-022-01668-9. 36307808 PMC9615186

[45] J. D. Wardyn , A. H. Ponsford , and C. M. Sanderson , “Dissecting Molecular Cross‐Talk Between Nrf2 and NF‐kappaB Response Pathways,” Biochemical Society Transactions 43 (2015): 621–626, 10.1042/BST20150014. 26551702 PMC4613495

[46] M. P. Gamcsik , M. S. Kasibhatla , S. D. Teeter , and O. M. Colvin , “Glutathione Levels in Human Tumors,” Biomarkers 17 (2012): 671–691, 10.3109/1354750X.2012.715672. 22900535 PMC3608468

